# Continuous flow synthesis of phosphate binding h-BN@magnetite hybrid material†

**DOI:** 10.1039/c8ra08336c

**Published:** 2018-12-05

**Authors:** Ahmed Hussein Mohammed Al-antaki, Xuan Luo, Alex Duan, Robert N. Lamb, Ela Eroglu, Wayne Hutchison, Yi-Chao Zou, Jin Zou, Colin L. Raston

**Affiliations:** Institute for Nanoscale Science and Technology, College of Science and Engineering, Flinders University Adelaide SA 5042 Australia colin.raston@flinders.edu.au; Centre for Marine Bioproducts Development, College of Medicine and Public Health, Flinders University Adelaide SA 5042 Australia; Trace Analysis for Chemical, Earth and Environmental Sciences (TrACEES), The University of Melbourne Victoria 3010 Australia; Department of Chemical Engineering, Curtin University Perth Australia; School of PEMS, University of New South Wales, ADFA Campus Canberra BC ACT 2610 Australia; Materials Engineering and Centre for Microscopy and Microanalysis, The University of Queensland Brisbane QLD 4072 Australia

## Abstract

Hexagonal boron nitride (h-BN) is rendered magnetically responsive in aqueous media by binding superparamagnetic magnetite nanoparticles 8.5–18.5 nm in diameter on the surface. The composite material was generated under continuous flow in water in a dynamic thin film in a vortex fluidic device (VFD) with the source of iron generated by laser ablation of a pure iron metal target in the air above the liquid using a Nd:YAG pulsed laser operating at 1064 nm and 360 mJ. Optimum operating parameters of the VFD were a rotational speed of 7.5k rpm for the 20 mm OD (17.5 mm ID) borosilicate glass tube inclined at 45 degrees, with a h-BN concentration at 0.1 mg mL^−1^, delivered at 1.0 mL min^−1^ using a magnetically stirred syringe to keep the h-BN uniformly dispersed in water prior to injection into the base of the rapidly rotating tube. The resulting composite material, containing 5.75% weight of iron, exhibited high phosphate ion adsorption capacity, up to 171.2 mg PO_4_^3−^ per gram Fe, which was preserved on recycling the material five times.

## Abstract 2

Vortex fluidic fabricated h-BN@magnetite under continuous flow in water exhibits recyclable high phosphate ion adsorption capacity.

## Graphical abstract

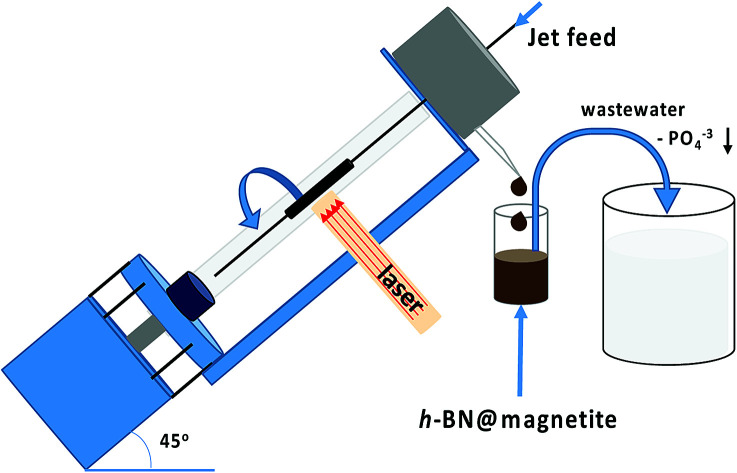

## Introduction

**Fig. 1:**
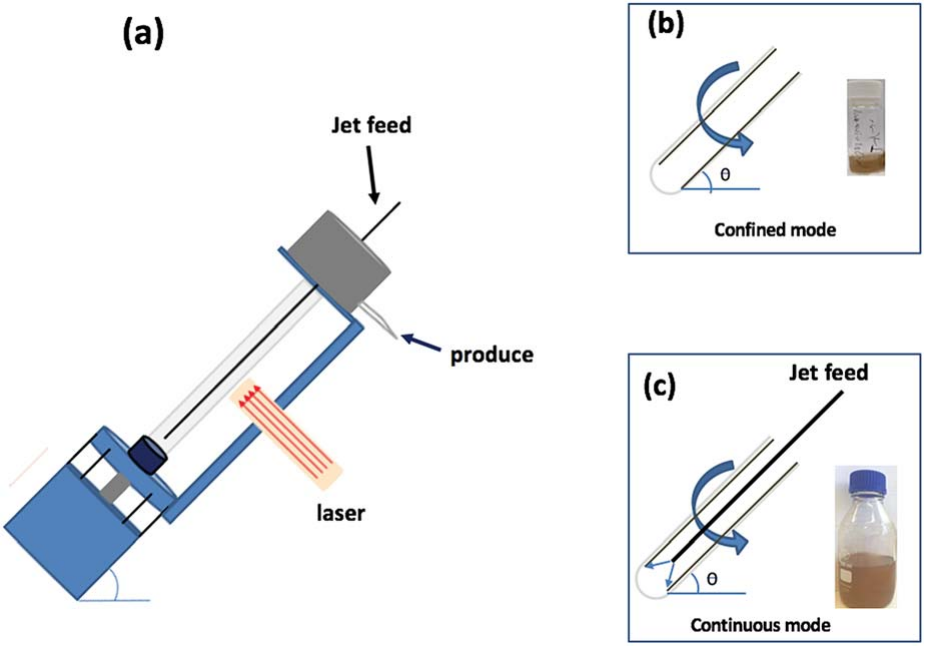
(a) Diagrammatic representation of the VFD used in association with a 5 nanosecond pulsed Nd:YAG laser operating at 1064 nm and 360 mJ, with a 8 mm diameter beam; the glass tube is rotated at 7.5k rpm and tilted at 45°. (b) Confined mode of operation of the VFD where a finite volume is placed in the rotating glass tube, for 15 min. (c) Continuous flow operation of the VFD with the flow rate of the liquid entering the rotating glass tube set at 1 mL min^−1^.

Polymorphs of boron nitride include the hexagonal arrangement of alternating sp^2^ hybridized boron and nitrogen atoms in 2D sheets, which is isostructural to graphene sheets in graphite, and this similarity in structure is attracting attention. However, in contrast to graphite, layered hexagonal boron nitride (h-BN) is transparent and is an insulator,^1^ and its dielectric and thermal properties make it useful for different electronic applications such as thermal interface materials for semiconductor packaging.^2,3^ In general, hexagonal carbon (h-C), where there are hexagonal six membered rings, can be metallic or semiconducting, depending on the dimensions and overall structure, whereas h-BN has a large band gap around 5.9 eV and the material is typically an insulator or indirect semiconductor.^4–6^ Electron energy loss spectroscopy (EELS) provides information on the number of layers in h-BN in a similar way to that of graphite.^7^ In contrast to graphene, h-BN has potential use as a vehicle for the delivery of protein and nucleobases.^8,9^ This arises from coordination of N or O donor groups in these macromolecules with electron deficient boron atoms in the h-BN. Such interactions are stronger than π–π interactions which prevail between graphene sheets or between carbon nanotube (CNTs) and the like.^10,11^ Multilayers of h-BN bind nanomaterials in imparting new properties, for example, magnetic response.^12,13^ The vortex fluidic device (VFD) is a thin film microfluidic platform which has a number of applications, including enhancing enzymatic reactions and organic synthesis, probing the structure of self-organised systems, protein folding, protein separation, exfoliation of 2D graphite and boron nitride, the fabrication of carbon dots, and more.^14–22^ The VFD has a borosilicate glass tubes (typically 20 mm in OD diameter, 17.5 mm ID diameter) open at one end, and is rotated at high speed (up to 9000 rpm) while inclined at an angle, from 0° to 90° relative to the horizontal position. The VFD can be used in two different modes of operations, (i) the confined mode for processing a finite volume of liquid in the rapidly rotating tube, and (ii) continuous flow mode where liquid is constantly fed into the tube, so that the processing can be scaled up, allowing the processing of large volumes by extending the operating time.^23^ The confined mode of operation of the VFD is often used as a proven starting point for optimizing a process, for fast tracking into continuous flow processing, Fig. 1.^24^ In further exploring the expanding applications of the VFD,^23,25^ we have investigated for the first time its use in preparing a composite material, containing both h-BN and superparamagnetic magnetite nanoparticles. The h-NB is decorated with magnetite nanoparticles *in situ* in the dynamic thin film in the VFD under continuous flow, under an atmosphere of air with the magnetite nanoparticles generated in a plume of iron formed by irradiating a pure iron metal block with a Nd:YAG pulsed laser, as previous reported for making exclusively aqueous solutions of superparamagnetic magnetite nanoparticles.^26^ The laser effectively creates a plasma plume over the surface of a pure iron target plate.^27–29^ We hypothesised that oxygen functionality on the surface of the magnetite nanoparticles would be effective in providing coordination interactions to B-atoms in h-BN, as well as providing a platform for binding phosphate (PO_4_^3−^) ions for waste water treatment.^30^

The dynamic thin film in the VFD imparts high mass transfer of particles into the liquid, and the high shear in the liquid is effective in generating small particles of magnetite, as well as some exfoliation of h-BN with then binding of the magnetite nanoparticles.^31^ The composite material, h-BN@magnetite, can be readily manipulated and confined in solution by a magnetic field, and we have established that it is effective as a novel absorbent in removing excess levels of phosphate from water resources, targeting its potential for avoiding environmental complications on ecosystems, such as eutrophication.^32^ Various techniques have already been applied for the removal of phosphorous in general from water bodies, as in biological processes and/or chemical adsorption by various adsorbing materials including activated alumina, ferric-oxides, various polymers, red-mud, sand, and zeolites.^33–37^ Iron oxides in general are effective for the removal of both phosphate and arsenate (AsO_4_^3−^) ions from the liquid effluents.^34^

## Experiments

### Materials

**Fig. 2:**
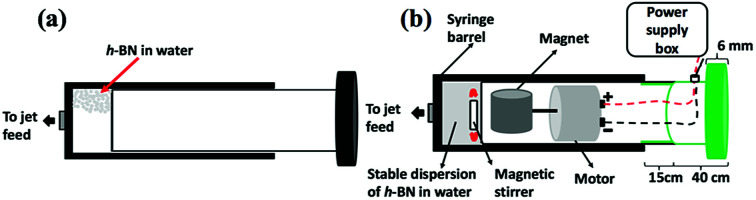
(a) Normal syringe. (b) Magnetic syringe is magnetics link with motor and sitting together inside the plunger which will affect onto magnetic bar sitting inside the syringe.

Hexagonal boron nitride (h-BN) powder flakes ∼1 μm in diameter with 99% purity were purchased from Sigma-Aldrich. High purity Fe (>99.998%) 8361 h iron rod, 5 mm thick (Koch-Light Laboratories) was used in the VFD as the laser target and Milli Q water was used for all the experiments. A suspension of h-BN in water was delivered at a controlled flow rate to the hemi-spherical base of the rapidly rotating tube. This was achieved using an in-house developed syringe pump featuring a motor housed in the plunger, for magnetically stirring the solution during delivery into the VFD tube, Fig. 2.

### Synthesis of h-BN@magnetite

**Fig. 3:**
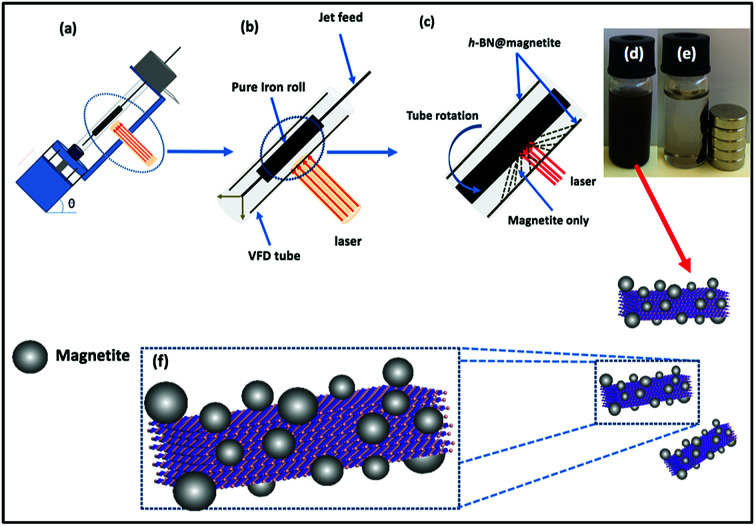
(a) VFD showing the position of the pure iron rod as a laser target being irradiated with a Nd:YAG pulsed laser operating at 1064 nm and 360 mJ, with the tube rotating at 7.5k rpm, tilt angle 45° and flow rate 1 mL min^−1^ for h-BN dispersed in water (0.1 mg mL^−1^). (b) Close up of the arrangement of the glass tube and position of laser irradiation. (c) Synthesis of the iron oxide nanoparticles (magnetite) attached to h-BN inside the glass tube. (d) Photograph of the solution exiting the VFD. (e) Photograph showing the effect of a magnet on h-BN@magnetite dispersed in water. (f) Cartoon of the h-BN@magnetite composite.

Magnetite nanoparticles were generated in the thin film in the VFD by irradiating an iron rod with a Nd:YAG pulsed laser (Quanta-Ray) operating at 1064 nm wavelength and 360 mJ, while a suspension of h-BN in water (at 0.1 mg mL^−1^) was delivered *via* a jet feed to the base of the tube. This was achieved using the above magnetically stirred solution of h-BN and water (from 1 L of a suspension of h-BN in water which was sonicated in a bath (6 KHz) for 25 minutes), at a flow rate of 1 mL min^−1^ which was controlled using a syringe pump, with the rotational speed of the tube in the VFD was set at 7.5k rpm and the tube angled at 45° relative to the horizontal position, Fig. 3.

### Characterisation

Hexagonal boron nitride (h-BN) decorated with magnetite nanoparticles was characterized using scanning electron microscopy (SEM) (Inspect FEI F50 SEM), atomic force microscopy (AFM) (Nanoscope 8.10 tapping mode), Raman spectroscopy (WiTec Alpha 300R *λ*_exc_ = 532 nm), X-ray photoelectron spectroscopy (XPS – Kratos Axis Ultra, with Monochromatic Al Kα X-ray source), X-ray diffraction (XRD) (Bruker D8 ADVANCE ECO, Co-Kα, *λ* = 1.79 Å), FTIR microscope (Nicolet iN10MX IR Microscope, Thermo Scientific), ATR-FTIR Perkin Elmer Frontier, TEM and HRTEM (FEI Tecnai F20 operated at 200 kV). Zeta potential (model: ZETASIZER Nanoseries nano-zs MALVERN) was also measured, along with iron analysis content using an atomic absorption spectrometer (GBC 933 plus) and Brunauer–Emmett–Teller (BET) surface area and porosity (micromeritics TriStar II). Magnetisation measurements were carried out using a Quantum design PPMS at 295 K in the field range ±1.50 T.

### Phosphate removal

Three different loadings of the magnetic h-BN adsorbent (10 mg; 25 mg; 50 mg) were separately mixed with phosphate containing artificial aquatic-media.^38^ (1.25 mL, which mainly includes phosphates, nitrates, carbonate buffer, micronutrients and vitamins at a buffered pH of 7.5.) Each mixture was then hand-vortexed (this not the VFD) for about 1 minute for initiating the experiments. The amount of phosphate ions remaining in the solution were monitored at the beginning and by the 30th minute according to our previous findings.^39,40^ The mixtures were centrifuged at 13 148*g* for 5 minutes and particle-free supernatants were collected for their spectrophotometric phosphate analysis by the colorimetric ascorbic acid method, or the so called orthophosphate method, which is a standard water-analysing procedure recognized by the United States Environmental Protection Agency.^41^ Phosphate analysing kits (HACH®, PhosVer® 3 phosphate reagent powder pillows) were employed before reading the phosphate concentration of the supernatant by a colorimeter (HACH® DR/870).

## Results and discussion

**Fig. 4:**
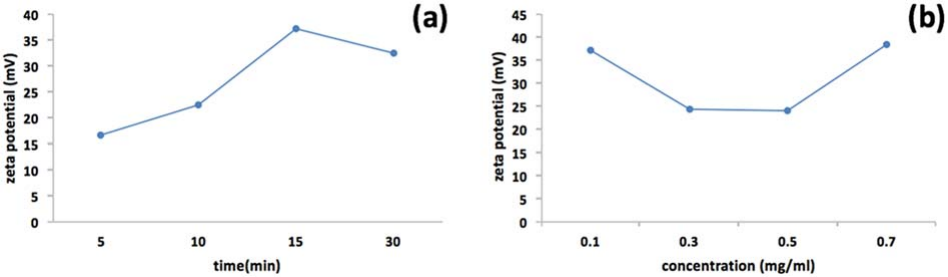
Zeta potential measurements for h-BN@magnetite prepared using the confined mode of operation of the VFD with the tube rotating at 7.5k rpm and tilted at 45°, with a pure iron rod irradiated using a pulsed laser operating at 1064 nm and 360 mJ, as a function of time, (a) with the concentration of h-BN at 0.1 mg mL^−1^, and (b) as a function of concentration of h-BN where the processing time was set at 15 min.

**Fig. 5:**
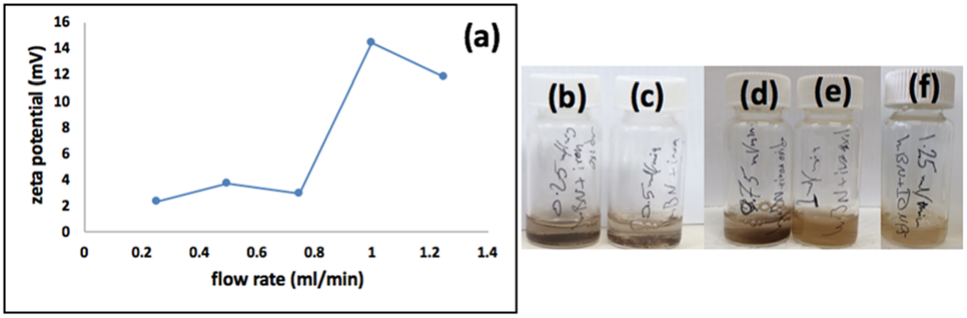
(a) Zeta potential for dispersions prepared under continuous flow, at flow rates of 0.25, 0.5, 0.75, 1, 1.25 mL min^−1^, with h-BN concentration at 0.1 mg mL^−1^, 7.5k rpm rotational speed, tilt angle 45° and laser operating at 1064 nm and 360 mJ. (b–f) Photographs of the dispersions prepared using flow rates of 0.25, 0.5, 0.75, 1 and 1.25 mL min^−1^ respectively.

**Fig. 6:**
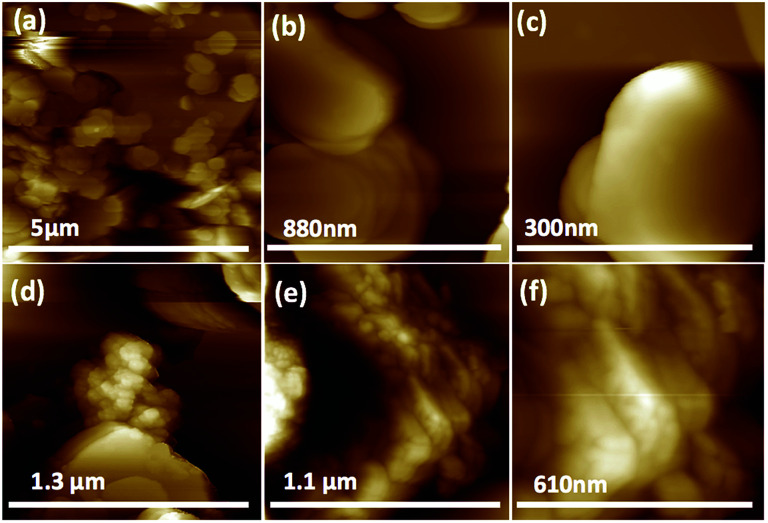
AFM images: (a–c) h-BN sheet as received. (d–f) h-BN@magnetite prepared at 7.5k rpm, tilt angle 45°, laser energy 360 mJ, the flow rate of h-BN dispersed in water (0.1 mg mL^−1^) 1 mL min^−1^.

**Fig. 7:**
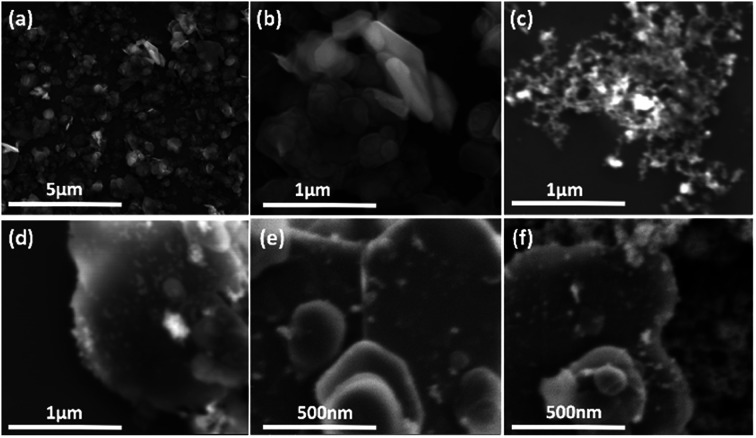
SEM images: (a and b) h-BN as received. (c) Magnetite formed in the absence of h-BN.^25^ (d–f) h-BN@magnetite; laser operation at 1064 nm and 360 mJ, rotational speed 7.5k rpm, and flow rate of h-BN disperse in water (0.1 mg mL^−1^) at 1 mL min^−1^.

We used a one-step VFD mediated process to prepare a new type of h-BN hybrid material directly from the 2D material and elemental iron, in the absence of any agents or harsh chemicals. This is a new use of the VFD, in generating metal oxide (magnetite) particles tethered to h-BN, with the stability of the material poised for use in different applications. Fig. 3 summarises the method of preparation of the h-BN@magnetite composite material within the dynamic thin film in a VFD with a pulsed laser operating at 1064 nm 360 mJ irradiating a fixed pure iron target above the liquid.^42^ Initially we used the confined mode of operation of the VFD to prepare the composite material, using zeta potential measurements to optimise the conditions for generating dispersions of high colloidal stability, along with SEM (see below). This indicates any build-up of magnetite nanoparticles on the surface of h-BN flakes.^43,44^ All processing was at a rotational speed of the tube in the VFD at 7.5k rpm, with the tube inclined at 45°, as the optimal parameters for a number of applications of the VFD.^24^ The pulsed laser was operated at 1064 nm and 360 mJ, as the optimal conditions for preparing magnetite nanoparticles in the absence of h-BN.^26^ Variation of processing time in the confined mode of operation of the VFD was first explored, with the volume of the liquid in the tube set at 1 mL, which was adhered to for all experiments using this mode, with the initial concentration of h-BN in water set at 0.1 mg mL^−1^. After 5 or 10 minutes post VFD processing the zeta potential was relatively low, below +25 mV, Fig. 4a, which is consistent with limited deposition of magnetite nanoparticles on the surface of h-BN. The zeta potential increased to +37.2 mV after 15 minutes of reaction which is consistent with colloidal stability of the particles in solution. After 30 minutes the zeta potential dropped, and thus 15 minutes processing time was deemed optimal in the confined mode. With the processing time set, the concentration of h-BN in water was then varied, Fig. 4b, with 0.3 and 0.5 mg mL^−1^ resulting in lower zeta potentials than for 0.1 and 0.7 mg mL^−1^. Suspensions of the composite material at 0.3 and 0.5 mg mL^−1^ were unstable (precipitating after a few mins) and thus zeta potential measurements were not possible. Consequently, 0.1 mg mL^−1^ of h-BN was deemed the optimised concentration under confined mode, and the processing was then translated into continuous flow mode of operation of the VFD. Zeta potentials for as prepared solutions at 0.25, 0.5, 0.75 and 1.25 mL min^−1^ flow rates were lower than 1 mL min^−1^, albeit not in the regime for colloidally stable material. Solutions prepared at flow rates of 0.25, 0.5 and 0.75 mL min^−1^ were indeed unstable, with h-BN@magnetite rapidly precipitating from solution. h-BN@magnetite prepared from a flow rate of 1.25 mL min^−1^ had a lower zeta potential, presumably reflecting a lower surface coverage of magnetite nanoparticles on the surface of the h-BN arising from a shorter resident time, Fig. 5. Thus there is a trade-off between optimising the flow rate for higher throughput *versus* the uptake of the magnetite nanoparticles onto the h-BN. To establish the size of the magnetite nanoparticles and how they reside on the surface of h-BN, AFM images were obtained. The topography of the surface of the h-BN before processing (as received) was smooth, Fig. 6a–c. For the composite material, patches of aggregated clusters, presumably magnetite nanoparticles, were evident on the outer surface of h-BN. The size of the particles making up these aggregates, Fig. 6d–f and S2.† SEM was also used to characterise the material, showing smooth surfaces before VFD processing and deposition of magnetite nanoparticles, Fig. 7a and b, with then randomly arranged clusters of particles on the surface post VFD processing, Fig. 7d–f and S3.† The presence of aggregated materials with its expected high surface area was in agreement with the result from Brunauer–Emmett–Teller (BET) experiments with the specific BET surface area increasing from 11.2 m^2^ g^−1^ for the as received h-BN to 91.0 m^2^ g^−1^ for the composite material, Fig. S4.† EDX of the composite material was consistent with the presence of h-BN and magnetite, Fig. S5.†

**Fig. 8:**
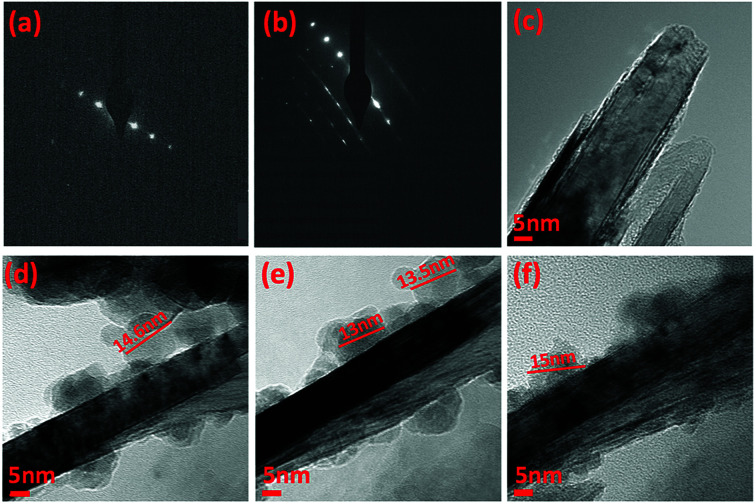
(a and c) SAED and HRTEM image of h-BN as received. (b and d–f) SAED and HRTEM images for h-BN@magnetite generated with the laser operating at 1064 nm and 360 mJ, rotational speed 7.5k rpm, and flow rate of h-BN dispersed in water (0.1 mg mL^−1^) at 1 mL min^−1^.

**Fig. 9:**
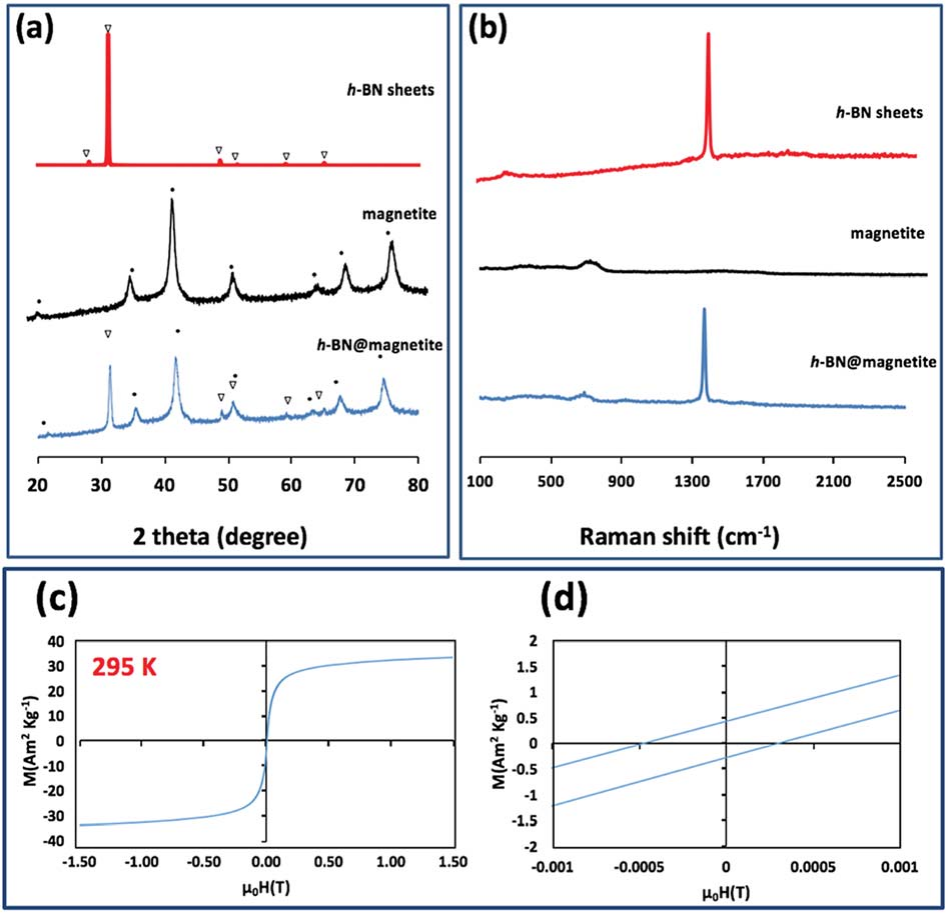
(a and b) X-ray diffraction and Raman spectra respectively of h-BN sheets as received, magnetite (in the absence of h-BN),^26^ and h-BN@magnetite. (c) Magnetization saturation for h-BN@magnetite. (d) Low-field interval of the magnetization curves of h-BN@magnetite; laser operating at 1064 nm and 360 mJ, rotational speed 7.5k rpm, and flow rate of h-BN disperse in water (0.1 mg mL^−1^) at 1 mL min^−1^.

**Fig. 10:**
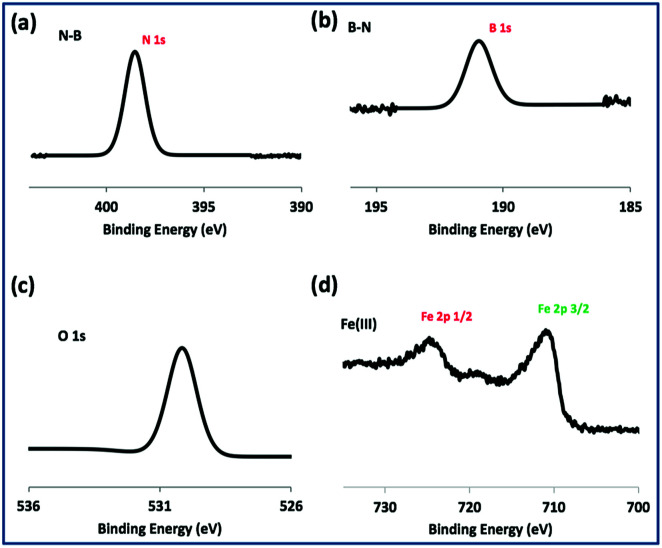
XPS spectra of h-BN@magnetite; laser operating at 1064 nm and 360 mJ, rotational speed 7.5k rpm, and flow rate of h-BN disperse in water 0.1 mg mL^−1^ at 1 mL min^−1^.

TEM-HRTEM images were used to determine the size and number of sheets of h-BN, Fig. 8c and S6a–c,† as well the size of the magnetite nanoparticles. The latter were determined to be from 8.5 nm to 18.5 nm, Fig. 8d–f and S6e,† which is consistent with the size of the particles generated in the absence of h-BN.^26^ Clearly the conditions studied using the VFD did not result in the formation of scrolls of h-BN which is possible under shear, at least for using the related spinning disc processing.^25^ In addition, there was little evidence of exfoliation of the h-BN, despite the VFD being effective for this, albeit in low percent conversion.^21^ The size of the h-BN sheets was from 150 nm to 3 μm which is similar to the as received material, with the inter planar spacing for the material 0.33 nm, Fig. S6f,† as expected. The size of the magnetite particles in h-BN@magnetite is consistent with the results from AFM and SEM. The presence of magnetite (IONPs) on h-BN sheets was confirmed using SAED, with SAED for as received h-BN in Fig. 8a, and that for h-BN@magnetite in Fig. 8b. The X-ray diffraction (XRD) pattern has 2*θ* peaks at 31.2 (002), 48.6 (100), 51.2 (101), 59.5 (102) and 64.8 (004) corresponding to h-BN sheets, and peaks at 21.2 (111), 35.5 (220), 41.8 (311), 50.8 (400), 63.4 (422), 67.7 (511) and 74.6 (440) corresponding to magnetite, Fig. 9a.^45–47^ The Raman spectrum has a peak at 1366 cm^−1^ which is consistent with the presence of h-BN, as the *E*_2g_ symmetry band,^48,49^ with a peak at 678 cm^−1^ corresponding to that expected for the presence of magnetite nanoparticles, Fig. 9b.^50^ According to the literature, magnetite nanoparticles <20 nm should be superparamagnetic, and this was indeed the case in determining the saturation magnetization (*M*) which is essentially constant at 34 A m^2^ kg^−1^Fig. 9c.^51,52^ In contrast, the saturation magnetization (*M*) of magnetite nanoparticles formed in the VFD in the absence of h-BN was 41 A m^2^ kg^−1^, where the size of the magnetite particles is similar.^26^ The reduction in saturation magnetization (*M*) of about 7 A m^2^ kg^−1^ in the composite material is consistent with attachment of the particles to the h-BN. In addition, the low-field interval of the magnetization curves show very little coercivity and remanence consistent with the presence of predominately superparamagnetic particles, Fig. 9d. ATR-FTIR and FTIR microscopy established a shift in both B–N stretching and B–N–B bending vibrations, respectively from 1348 cm^−1^ in h-BN to 1372 cm^−1^ in the composite material, and 768 cm^−1^ to 800 cm^−1^, Fig. S7.†^53,54^ In addition, the spectrum of h-BN@magnetite has a peak at 624 cm^−1^ corresponding to Fe–O–Fe vibrations in magnetite, Fig. S7.†^55^ Comparing XPS data for as received h-BN and h-BN@magnetite, Fig. 10a and b, clearly establishes the presence of magnetite in the latter, with binding energies peaks for Fe 2p_1/2_ and Fe 2p_3/2_ at 724.4 eV and 710.7 eV respectively (Fig. 10d).^56–58^ The main XPS peak corresponding to oxygen atoms in magnetite are at 530.1 eV, Fig. 10c.^46^

Phosphate removal efficiency of the material were tested by applying three different loading concentrations of the identical h-BN@magnetite sample as the adsorbent (10 mg; 25 mg; 50 mg), which were separately mixed with phosphate containing artificial aquatic-media having an initial phosphate concentration of 23.2 ± 0.15 mg L^−1^, which is in the range of a typical sewage and urban wastewaters with 10–30 mg L^−1^ phosphate levels.^59^ According to our previous findings with superparamagnetic imposed diatom frustules^39^ and mesocellular siliceous foams impregnated with iron oxide,^40^ the 30^th^ minute was chosen as the optimal time-interval for measuring the phosphate removal capacity of the adsorbent.

**Fig. 11:**
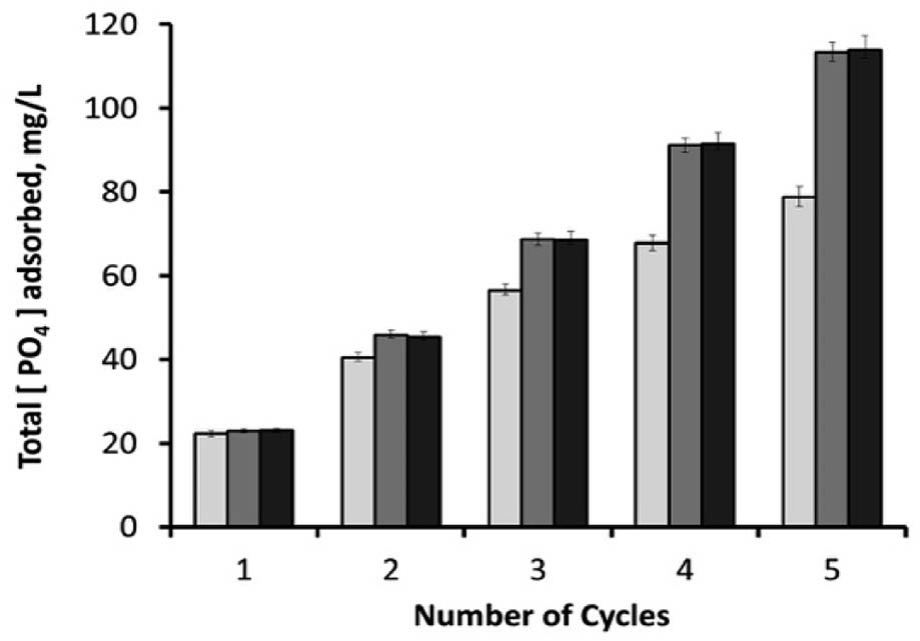
Cumulative amount of PO_4_^3−^ adsorbed in mg L^−1^ after each sequential cycle, for three different loadings of magnetic h-BN samples: (1) 10 mg adsorbent (light-grey columns); (2) 25 mg adsorbent (dark-grey columns); and (3) 50 mg adsorbent (black columns).

After 30 minutes, the initial phosphate concentration (23.2 ± 0.15 mg L^−1^) dropped to around 0.9 mg L^−1^ (96.1% removal efficiency) with 10 mg adsorbent; 0.2 mg L^−1^ (99.1% removal efficiency) with 25 mg adsorbent; and 0.15 mg L^−1^ (99.4% removal efficiency) with 50 mg adsorbent. Used samples were then washed with deionized water through hand-vortexing for 1 minute, followed by the removal of supernatant after its centrifugation at 13 148*g* for 5 minutes. Remaining samples were then mixed again with phosphate-containing fresh media (1.25 mL), and the phosphate content of the consecutive cycle was analysed after 30 minutes. As shown in Fig. 11, the increase in adsorbent loading from 10 mg to 25 mg had a positive effect on the total amount of PO_4_^3−^ adsorbed during consecutive cycles. It was also observed that higher loadings of adsorbent (25 and 50 mg) had similar phosphate adsorption efficiencies, revealing the sufficiency of 25 mg adsorbent for this process. By the end of the 5^th^ consecutive cycle, 50 and 25 mg of adsorbents yielded an overall phosphate adsorption value of around 113.5 mg L^−1^, which was followed by 78.8 mg L^−1^ by a 10 mg sample. Recycling the material for sequential mechanical-mixing and washing steps has been proven to be highly effective for the reutilization of the magnetic h-BN material as an adsorbent, especially for the highest adsorbent loadings (25, and 50 mg) that particularly sustained their adsorption capacities. On the other hand, 10 mg adsorbent showed lower recovery compared to the higher loadings after each recycling step (Fig. 11 and S11†), revealing that the active sites of magnetic nanoparticles on the surface started to get saturated with phosphate ions. Due to the fact that the iron oxide component is mainly responsible for the phosphate removal,^34,39,40^ converting the phosphate removal values into their adsorption capacities revealed a reverse correlation between the overall phosphate adsorption capacity and the adsorbent loading concentration. Since the material has an iron content of 5.75% (w/w) (atomic absorption spectroscopy), 10 mg sample yielded an overall adsorption capacity of 171.2 mg PO_4_^3−^ per gram Fe, followed by 98.5 mg PO_4_^3−^ per gram Fe for 25 mg sample, and49.5 mg PO_4_^3−^ per gram Fe for 50 mg sample. These values are within the higher range of the other literature data reported for the various types of adsorbents containing iron oxide nanoparticles.^39,40,59^ In our previous studies, superparamagnetic imposed diatom frustules achieved an overall adsorption capacity of around 45 mg PO_4_^3−^ per gram Fe,^39^ and mesocellular siliceous foams impregnated with iron oxide showed an overall adsorption capacity of around 79.2 mg PO_4_^3−^ per gram Fe.^40^ High adsorption capacities of the current study reveal the suitability of h-BN structure as an ideal frame for the active sites of magnetic nanoparticles.

## Conclusion

The as-synthesis new composite h-BN@magnetite is directly available as a one-step synthesis, using a VFD, without the need for adding any other materials or potentially toxic solvent. The superparamagnetic magnetite nanoparticles in h-BN@magnetite are dispersed randomly on the h-BN surface, with the composite material having high porosity, with a surface area of 91.03 m^2^ g^−1^. The surface lends itself for binding functional materials, including catalysts,^55^ and for application in waste water treatment. This has been established in binding of phosphate in waste water, with the h-BN@magnetite being readily collected using a magnetic field. Also significant is that the phosphate adsorption capacity is up to 171.2 mg PO_4_^3−^ per gram of Fe, and the materials can be readily recycled.

## Conflicts of interest

There are no conflicts to declare.

## Supplementary Material

RA-008-C8RA08336C-s001
