# Supplementary material for: Continuous flow synthesis of phosphate binding h-BN@magnetite hybrid material
Source: RSC Adv. 2018 Dec 5;8(71):40829–35. doi: 10.1039/c8ra08336c (PMC9091421; doi:10.1039/c8ra08336c)
Supplement: RA-008-C8RA08336C-s001 [file RA-008-C8RA08336C-s001.pdf]

Electronic Supplementary Material (ESI) for RSC Advances.
This journal is © The Royal Society of Chemistry 2018

Continuous flow synthesis of phosphate binding *h*-BN@magnetite hybrid material

Ahmed Hussein Mohammed Al-antaki [a], Xuan Luo [a, b], Alex Duan [c], Robert N. Lamb [c], Ela Eroglu [d], Wayne Hutchison [e], Yi-Chao Zou [f], Jin Zou [f], Colin L. Raston [a*]

[a.] *Institute for Nanoscale Science and Technology, College of Science and Engineering, Flinders University, Adelaide, SA 5042, Australia.*
[b.] *Centre for Marine Bioproducts Development, College of Medicine and Public Health, Flinders University, Adelaide, SA 5042, Australia.*
[c.] *Trace Analysis for chemical, Earth and Environmental Sciences (TrACEES), the university of Melbourne, Victoria 3010, Australia.*
[d.] *Department of Chemical Engineering, Curtin University, Perth, Australia.*
[e.] *School of PEMS, University of New South Wales, ADFA campus, Canberra BC, ACT 2610, Australia.*
[f.] *Materials Engineering, and Centre for Microscopy and Microanalysis, The University of Queensland, Brisbane, QLD 4072, Australia.*

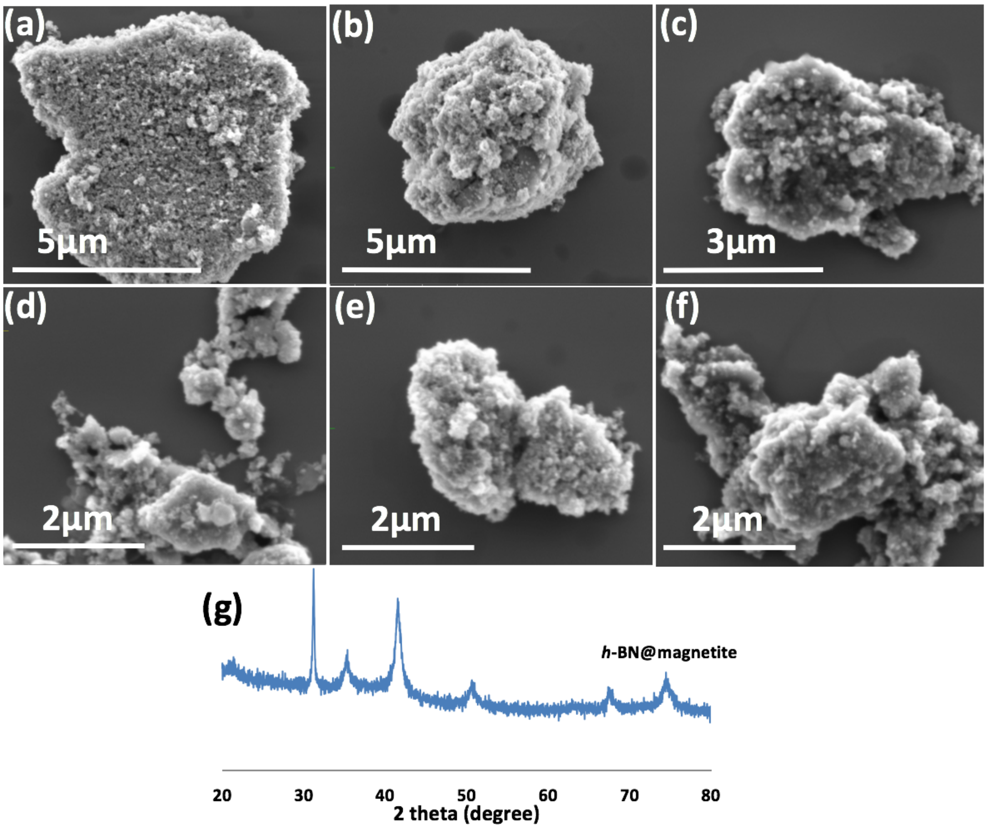

Figure S1. *h*-BN@magnetite composite prepared using the confined mode of operation of the VFD for 15 min, with pulsed laser operating at 1064 nm and 360 mJ, irradiating a pure iron target. Rotational speed at 7.5k rpm for a 20 mm OD glass tube, tilt angle 45º and h-BN dispersed in water (0.1 mg/mL). (a-f) SEM images and (g) XRD pattern.

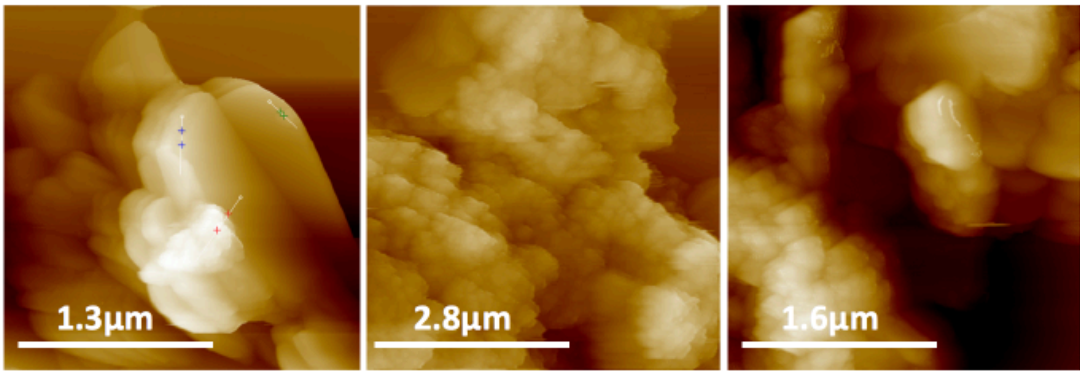

Figure S2. AFM images for *h*-BN@magnetite composite prepared in a VFD under continuous flow mode with a pulsed laser operating at 1064 nm and 360 mJ, irradiating a pure iron target. Rotational speed at 7.5k rpm, tilt angle 45º and flow rate 1 mL/min.

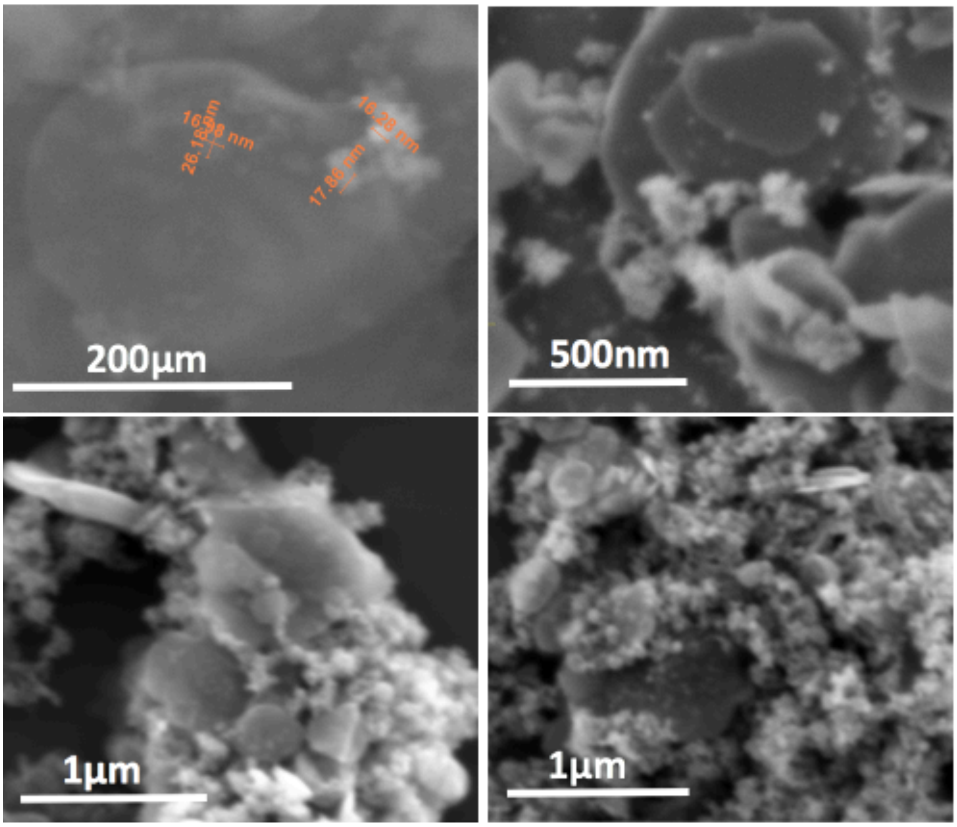

Figure S3. SEM images of *h*-BN@magnetite composite prepared under continuous flow mode with a pulsed laser irradiating a pure iron target, operating at 1064 nm and 360 mJ, with the 20 mm OD glass tube rotating at 7.5k rpm, tilt angle 45° and flow rate at 1 mL/min.

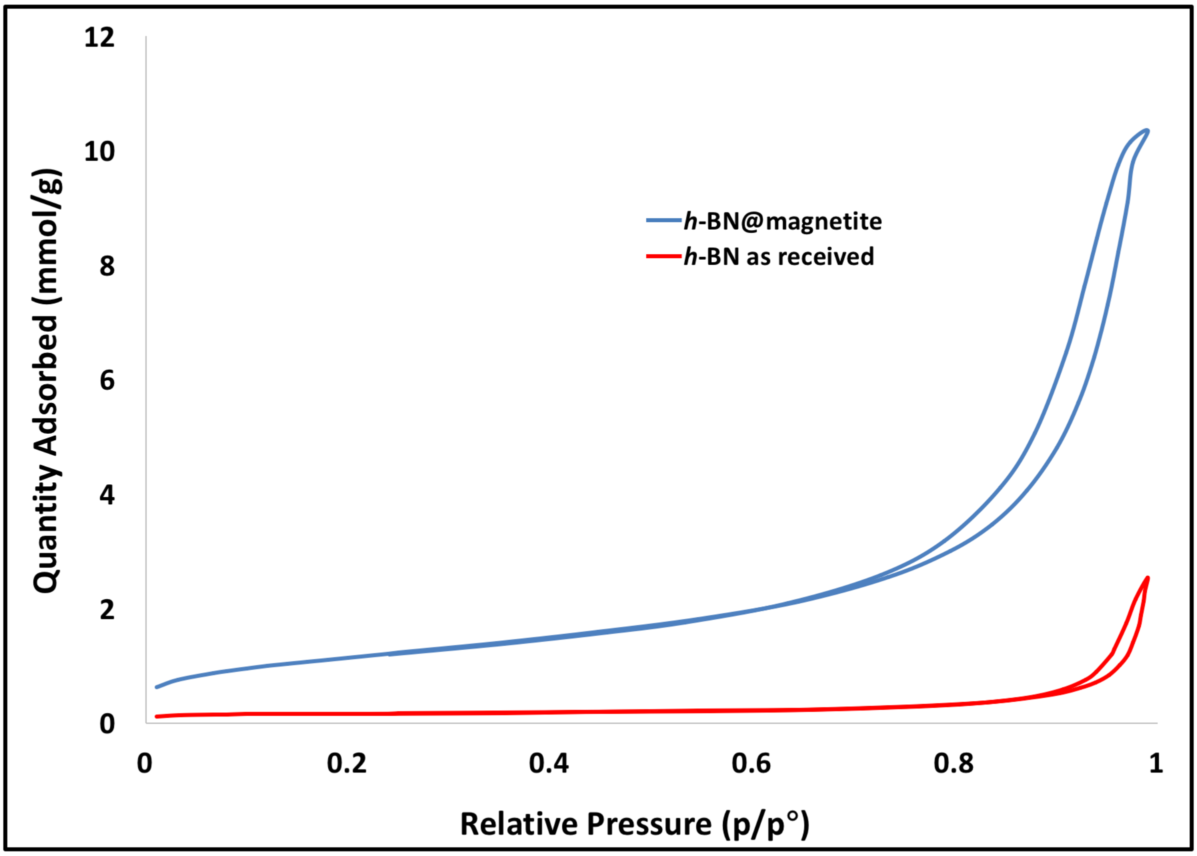

Figure S4. BET chart (blue) of *h*-BN@magnetite prepared under continuous flow with a pulsed laser irradiating a pure iron target, operating at 1064 nm and 360 mJ, with the VFD tube 20 mm in diameter rotating at 7.5k rpm, tilt angle 45º and flow rate at 1 mL/min; (red) as received *h*-BN.

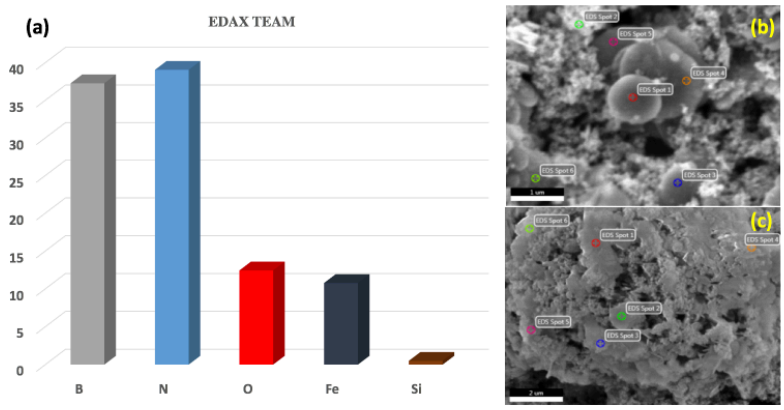

Figure S5. (a) EDX data for elemental analysis in b and c. (b and c) SEM images at various locations of *h*-BN@magnetite composite prepared under continuous flow with a pulsed laser irradiating a pure iron target operating at 1064 nm and 360 mJ, with a glass tube 20 mm in diameter rotating at 7.5k rpm, tilt angle 45º and flow rate at 1 mL/min.

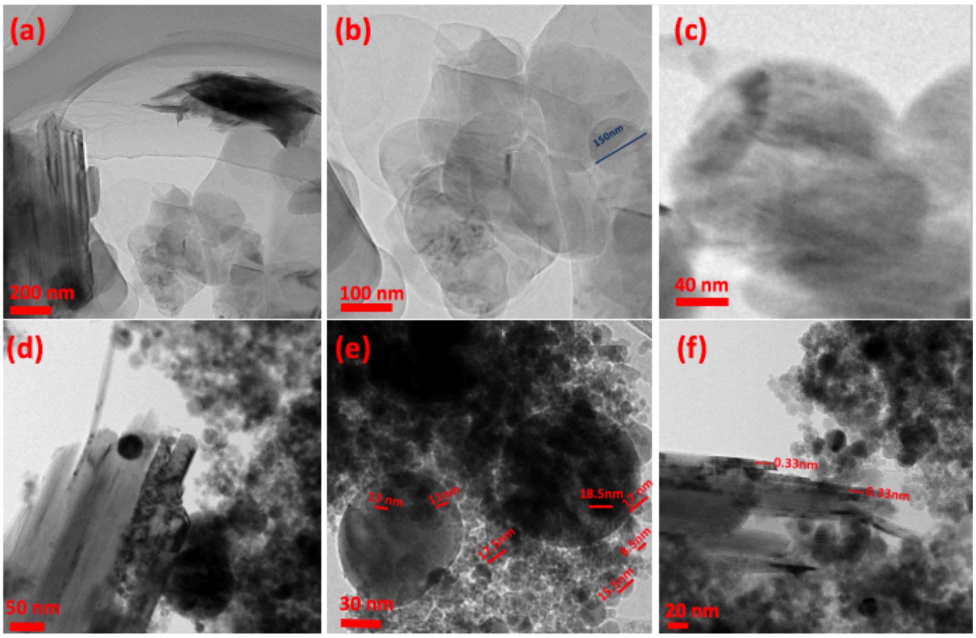

Figure S6. HRTEM images (a-c) as received *h*-BN, (d-f) *h*-BN@magnetite composite prepared using a pulsed laser irradiating a pure iron target operating at 1064 nm and 360 mJ, with a 20 mm glass tube rotating at 7.5k rpm, tilt angle 45º and flow rate at 1 mL/min.

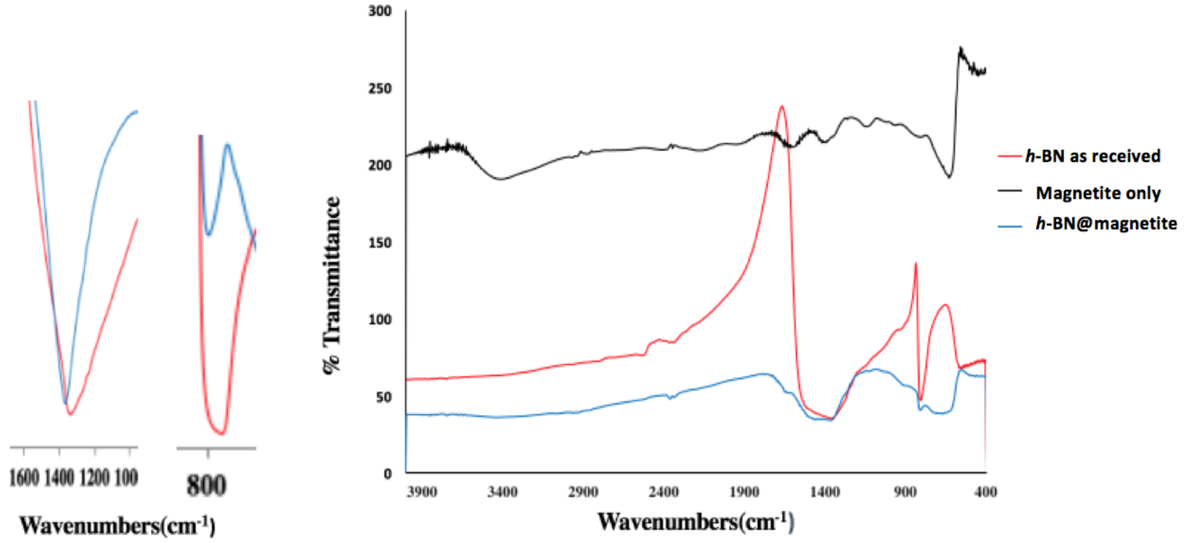

Figure S7 . ATR FTIR spectra for as received *h*-BN and *h*-BN@magnetite composite compared with magnetite prepared with a pulsed laser irradiating a pure iron target, operating at 1064 nm and 360 mJ with a 20 mm OD tube rotating at 7.5k rpm, tilt angle 45º and a flow rate of water at 0.1 mL/min.[1] The *h*-BN@magnetite was prepared as for the magnetite but with *h*-BN dispersed in water (0.1 mg/mL) at a flow rate of 1 mL/min.

Control experiments: Three control experiments were undertaken to decouple any effect of the laser on the *h*-BN and shear stress in the dynamic thin film in the VFD, with the characterization of the material using SEM and XRD, and zeta potential.

1- **Processing *h*-BN in the VFD in the absence of a pulsed laser.** *h*-BN dispersed in water (0.1 mg/mL) was delivered via a jet feed to the base of a glass tube 20 mm in diameter using a jet feed, with the flow rate at 1 mL/min, rotational speed 7.5k rpm and tilt angle 45°. The color of the processed liquid was similar to the pre-processed sample, with the zeta potential similar at -19.7mV.

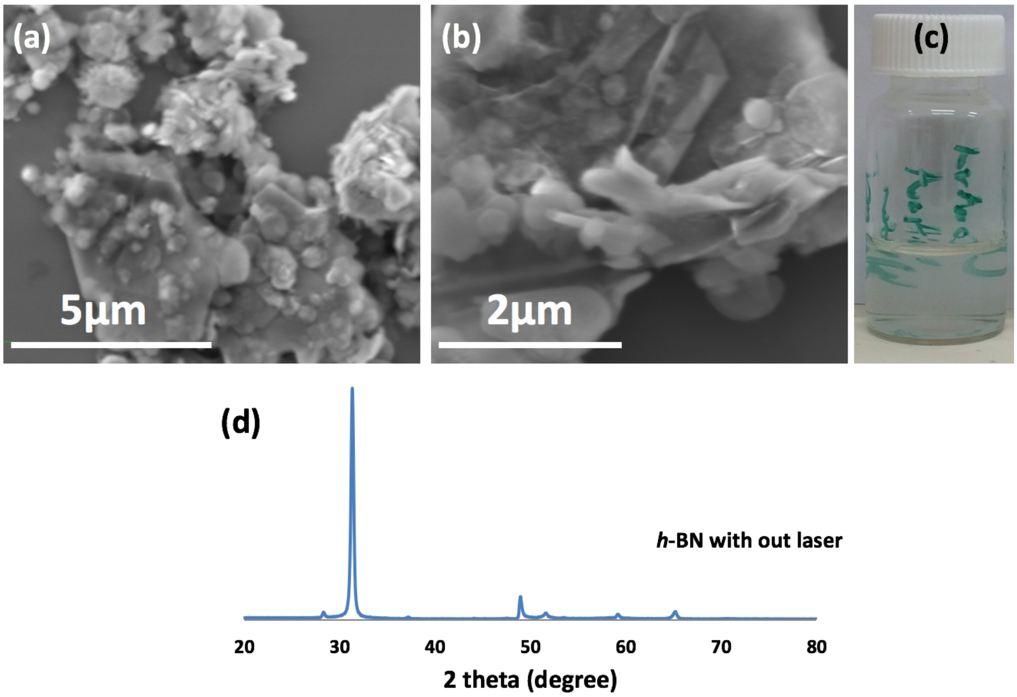

Figure S8. (a, b) SEM images for *h*-BN processed in the VFD under continuous flow with a flow rate of a dispersion of *h*-BN in water (0.1 mg/mL) at 1 mL/min, rotation speed 7.5k rpm, tilt angle 45° in the absence of a laser. (c) Photograph of the dispersion of *h*-BN solution after processing. (d) XRD of the *h*-BN after processing.

2- **Processing with the pulsed laser irradiating the solution without irradiating the iron target**. A dispersion of as received *h*-BN in water (0.1mg/mL) was delivered to the base of a 20 mm OD glass tube at 1 mL/min, with the tube rotating at 7.5k rpm and at a tilt angle of 45°; the pulsed laser was operated at 1064 nm and 360 mJ. The color of the processed

liquid was similar to the pre-processed sample, with a similar zeta potential value at -18.4mV.

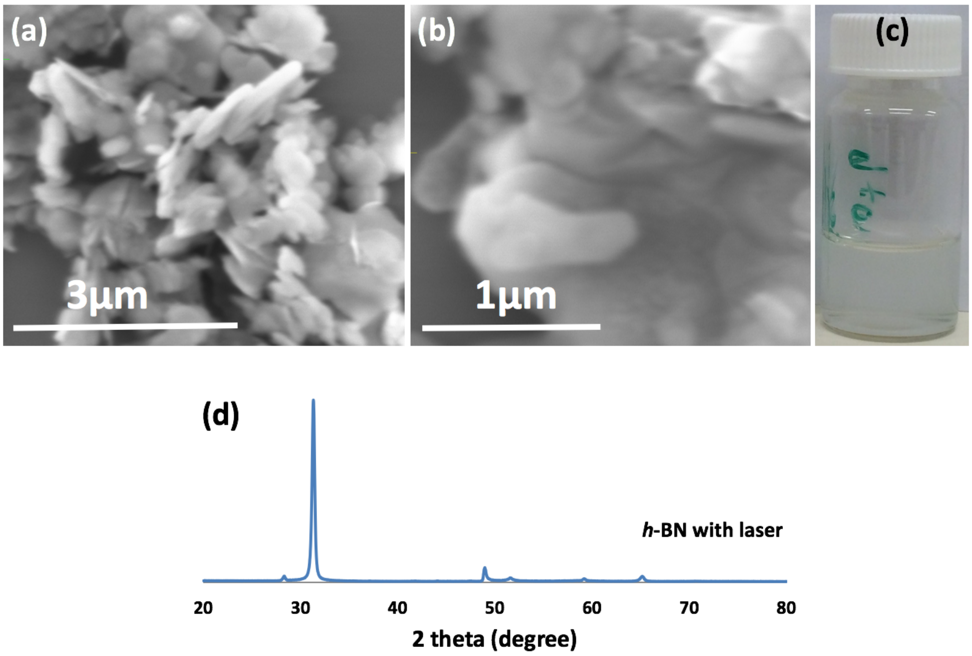

Figure S9. (a, b) SEM images for *h*-BN processed in a VFD, with a dispersion of the as received material delivered to the base of a 20 mm OD glass tube rotating at 7.5k rpm, at a flow rate of a dispersion of *h*-BN in water (0.1 mg/mL) at 1 mL/min for a tilt angle of 45º and the pulsed laser operating at 1064 nm and 360 mJ, without the laser irradiating the iron rod. (c) Photograph of the processed dispersion of *h*-BN. (d) XRD of *h*-BN after processing.

3- **Confined mode processing of a mixture of *h*-BN with IONPs (magnetite nanoparticles).** A mixture of *h*-BN dispersed in water (0.1 mg/mL) and preformed magnetite particles (0.5 mg/mL) previously prepared in the VFD using our recently reported procedure for the exclusive formation of this material.[1] The total volume of liquid in the tube was 1 mL, with the rotational speed set at 7.5k rpm, the tilt angle at 45°, and the processing time at 15 mins, in the absence of a laser. The color of the resulting solution was brown, as expected, and the SEM images similar to *h*-BN@magnetite composite prepared *in situ*. However, the zeta potential had two peaks, -8.03 mV and -17.3mV, presumably the former corresponding with some of the *h*-BN particles devoid of surface bound magnetite particles, noting that the latter is close to zeta potential for as received *h*-BN (-19.7mV). In contrast the zeta potential for *h*-BN@magnetite prepared directly in the presence of a laser is + or -?37.2 mV.

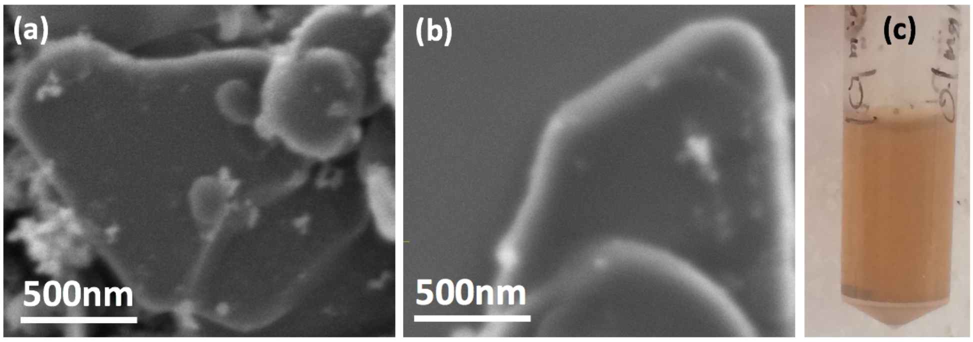

Figure S10. (a, b) SEM images for the material formed on processing 1 mL of a mixture of *h*-BN dispersed in water (0.1 mg/mL) and magnetite nanoparticles (0.5 mg/mL), using the confined mode of operation of the VFD (15 mins, 45° tilt angle, 7.5k rpm rotational speed, 20 mm OD glass tube), in the absence of laser irradiation. The magnetite nanoparticles (12 nm in diameter) where prepared using our published procedure in the VFD.[1] (c) Photograph of the *h*-BN@magnetite solution after processing under these conditions.

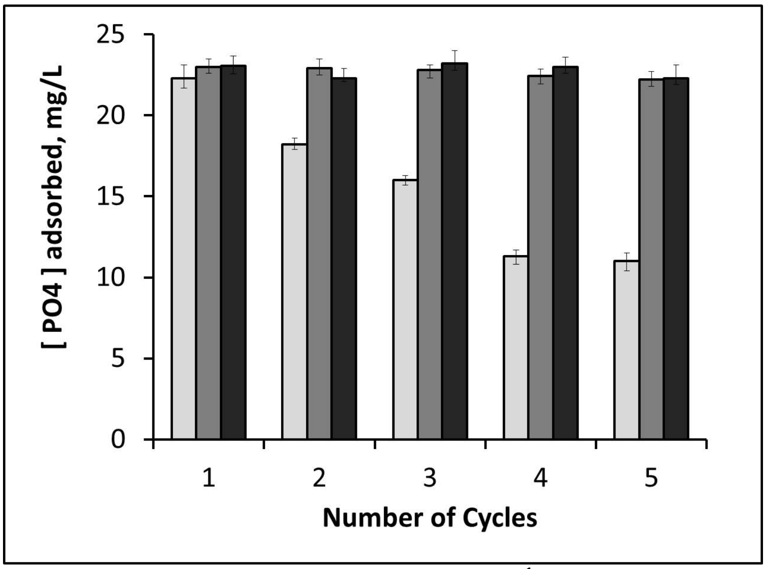

Figure S11. Individual amount of PO43- adsorbed in mg L^{-1} by each sequential cycle, for three different loadings of magnetic *h*-BN samples: (1) 10 mg adsorbent (light-grey columns); (2) 25 mg adsorbent (dark-grey columns); and (3) 50 mg adsorbent (black columns).

1. X. Luo, A. H. M. Al-Antaki, T. M. D. Alharbi, W. D. Hutchison, Y.-c. Zou, J. Zou, A. Sheehan, W. Zhang and C. L. Raston, *ACS Omega*, 2018, **3**, 11172-11178.
